# Chronic Subdural Hematoma (CSH) is Still an Important Clinical Problem. Analysis of 700 Consecutive Patients

**DOI:** 10.1515/tnsci-2019-0042

**Published:** 2019-11-06

**Authors:** Zbigniew Kotwica, Agnieszka Saracen, Ireneusz Dziuba

**Affiliations:** 1 Department of Neurosurgery, Faculty of Health Sciences and Physical Education, Kazimierz Pulaski University of Technology and Humanities, Radom, Poland

**Keywords:** chronic subdural hematoma, craniotomy, burr holes, closed system drainage, anticoagulants

## Abstract

**Background:**

Chronic subdural hematoma (CSH) is still an important neurosurgical problem and the number of patients increases despite the progress in early diagnosis of cerebral lesions.

**Methodology:**

We analyzed a group of 700 consecutive patients treated in neurosurgical departments for CSH. Clinical state on admission was evaluated according to the Markwalder scale, all patients had CT studies and were operated using craniotomy or burr holes with closed system drainage techniques.

**Results:**

More than 50% had extensive intracranial bleeding, almost half of the patients were treated with oral anticoagulants. The patients with extensive fresh bleeding were in significantly worse states on admission and were treated by craniotomy and external capsulectomy (42%). All the others had burr holes and closed system drainage of the subdural space. Results of treatment were acceptable, 2% died, and 1.5% remained vegetative, due to massive hemorrhage and severe neurological deficits on admission.

**Conclusions:**

Despite a progress in diagnosis, CSH still remains an often cause of severe intracranial complications. The rising number of occurrences of this lesion is strictly connected with a wide use of oral anticoagulants. Surgical removal of CSH still remains the best type of treatment for such lesions.

## Introduction

Chronic subdural hematoma (CSH) is defined as encapsulated hematoma having an outer and inner membrane [1, 2]. This kind of hematoma occurs mainly in older populations, and if not treated surgically leads to death [3, 4]. In many patients CSH is slowly developing after mild head trauma, however the number of patients with no trauma history is rising, probably due to anticoagulant therapy [3, 5, 6, 7].

In this paper we analyzed results of surgical treatment of CSH.

## Material and methods

We analyzed a group of 700 consecutive patients treated for CSH from 2003 to 2017. There were 410 males and 290 females, 42 to 94 year old, with a mean age 76 years. Table 1 shows the age and sex of patients.

**Table 1:** An age and sex of patients treated for CSH

Sex Age (years)	Male	Female	Total
40-50	3	0	3
51-60	16	1	17
61-70	86	74	160
71-80	87	89	176
81-90	216	112	328
> 90	2	14	16
Total	410	290	700

**Figure 1:**
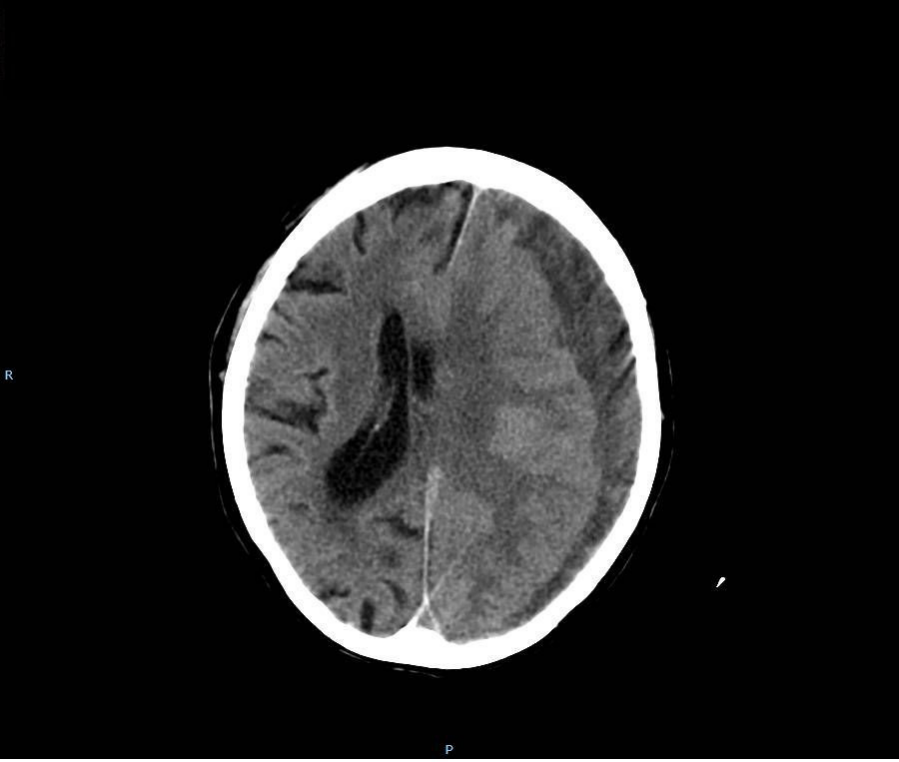
hypodense (typical) chronic subdural hematoma

**Figure 2:**
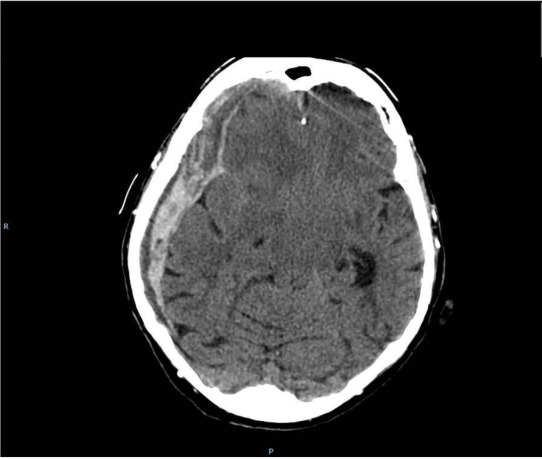
chronic subdural hemtoma with fresh bleeding to the hematoma cavity

From 700 patients a history of head trauma was noted in 386 patients (55%) with a delay from 2 weeks up to 4 months. 336 patients (48%) received anticoagulants, in 74 of them INR was above 3,0, and in 12 patients was higher than 6.0. On admission the patients were evaluated according to the Markwalder scale [1] and diagnosed with brain CT. CT revealed in all patients the presence of subdural hematoma (figure 1), in 78% fresh blood was noted, and in 294 (42%) patients with fresh extensive bleeding (figure 2). These last patients were treated by craniotomy, evacuation of hematoma with extensive capsulectomy [4, 5, 6], all the others were treated by burr holes and closed system drainage [2, 5, 8, 9]. In 24 patients bilateral chronic subdural hematoma was diagnosed and all these patients were treated by bilateral burr holes with closed system drainage. After CT diagnosis the patients were operated under general anesthesia. The patients with prolonged INR were previously treated with intravenous prothrombin complex concentrate and fresh- frozen plasma [10]. After initial surgery routine control CT was done 3 to 5 days later.

Results of treatment were evaluated according to GOS [11] 30 days after surgery.

## Results

On admission 126 patients (18%) were comatose, and 392 patients (56%) presented severe neurological deficit which developed within 0 to 7 days before admission. The patients with fresh bleeding into the hematoma cavity were significantly clinically worse than patients with hypodense subdural lesion. Extensive fresh bleeding was noted in 294 patients (42%) and all these patients were treated by craniotomy with external capsulectomy. The patients with fresh bleeding received oral anticoagulants significantly more frequently. Table 2 shows clinical state of patients in Markwalder scale [1] compared to the presence or absence of fresh bleeding into hematoma cavity.

**Table 2:** Clinical state of patients in Markwalder scale compared to the presence or absence of fresh bleeding into hematoma cavity.

Clinical state in Markwalder scale Fresh bleeding	I	II	III	IV	Total
Noted	1(1)	54(16)	366(205)	125 (92)	546(314)
Absent	45(9)	82(6)	26(6)	1(1)	154(22)
Total	46(10)	136(22)	392(211)	126(93)	700(336)

(number of patients receiving oral coagulants)

From 700 patients, postoperative course was uneventful in 570 and they were discharged from the ward 5 to 10 days after hematoma evacuation. From 130 remaining patients 26 had hematoma recurrence requiring repeated surgery procedure – 16 treated by burr holes and 10 with craniotomy; 28 patients developed bronchopneumonia, 73 presented symptoms of postoperative pneumocephalus, which disappeared in all of them within 5 to 10 days after surgery. 16 patients (2%) died.

Table 3 shows the results of treatment in comparison with the type of surgical procedure, table 4 shows results of treatment according to the clinical state on admission

**Table 3:** Results of treatment in comparison with the type of surgical procedure.

Results of treatment in GOS Type of surgery	I	II	III	IV	V	Total
Burr holes and closed system drainage	1	1	217	126	61	406
Craniotomy with capsulectomy	15	11	179	56	33	294
Total	16	12	396	182	94	700

**Table 4:** Results of surgical treatment according to the clinical state on admission.

GOS Markwalder Scale on admission	I	II	III	IV	V	Total
I	0	0	0	16	30	46
II	0	0	20	76	40	136
III	6	4	277	82	23	392
IV	10	8	99	8	1	126
Total	16	12	396	182	94	700

Results of treatment mainly depended on the clinical state on admission. All deaths were noted in comatose or severe neurological deficit patients. Age and sex of patients had no influence on surgical results. Worse results of treatment were noted in patients treated by craniotomy, however all these patients were in worse clinical state before surgery and extensive skull opening was decided because of the extent of fresh intracranial bleeding.

The patients requiring anticoagulants received low molecular weight heparin in therapeutic doses, and if the control CT performed 3-5 days after surgery did not reveal hematoma recurrence, they returned to oral anticoagulant therapy.

## Discussion

Chronic subdural hematoma is still common in neurosurgical practice. The number of patients with such lesion does not reduce, and comparing to previous literature data, initial clinical status on admission is worse than 25 years ago [2, 6, 8]. Significant fresh bleeding into hematoma cavity was noted in 78% of the patients, 48% received oral anticoagulants. This last group presented extensive intracranial bleeding, even with therapeutic INR values.

Wide use of oral anticoagulants, especially in older populations raises the number of intracranial spontaneous bleeding, both intracerebral [12] as well as subdural [4, 5, 6, 7]. Minimal repeated hemorrhages slowly leads to the development of CSH, and in many patients such course of illness is complicated by extensive subdural hemorrhage even causing brain herniation [6]. Burr holes or twist drill craniostomy followed by closed system drainage of subdural space is a method of choice in the treatment of this lesion [2, 9, 13]. We used it in all patients with no CT signs of severe fresh bleeding. However 42% of patients required craniotomy due to large subdural deposit of fresh blood and significant intracranial shift. These patients were clinically significantly worse than patients with no extensive fresh bleeding.

Despite the fact that 74% of patients on admission presented severe neurological Translational Neuroscience deficits and deep consciousness disturbances, the results of treatment were good. We noted 16 deaths (2%), and 12 (1.5%) patients remained in a vegetative state. All the others improved after surgery.

The number of recurrences was acceptable – 3.5% required repeated surgery. All patients had control CT 3 to 5 days after initial surgery, however it does not seem necessary [14], and control CT should be performed only in patients, whose clinical status worsens after initial surgical treatment. In 73 (10.5%) patients control CT showed significant pneumocephalus, however no one patient presented clinical symptoms or required surgical treatment due to this complication. In all patients, CT performed during the first week after surgery shows blood deposit in the subdural space, but it does not require any intervention. If the patient improves clinically no procedures should be performed. Bilateral hematomas were noted in 3.5%, however all these patients were in good clinical condition and were treated with bilateral burr holes [15].

It must be pointed out, that CSH is still a very common intracranial lesion. Recovery after neurosurgical treatment is good in most patients, however poor outcomes are noted in 2-4%, mainly due to extensive fresh bleeding into hematoma cavity. Wide use of oral anticoagulants raises the number of intracranial iatrogenic complications and it should be in mind of the medical staff, that this group of patients easily develops intracranial subdural bleeding, which in most of them requires surgical treatment. Nonsurgical treatment is, in our opinion, not acceptable. It lasts for a long time, and requires a long pharmacological treatment, which especially in older patients can lead to significant extracerebral complications [16, 17].
